# Quelle stratégie chirurgicale adopter devant une hémorragie du post-partum et comment améliorer les résultats de la ligature des artères hypogastriques?

**DOI:** 10.11604/pamj.2016.25.96.9242

**Published:** 2016-10-18

**Authors:** Mehdi Kehila, Sadok Derouich, Dalenda Chelli, Omar Touhami, Sofiene Ben Marzouk, Sonia Ben Khedher, Mohamed Bedis Chanoufi, Fethia Boudaya

**Affiliations:** 1 Service C, Centre de Maternité et de Néonatologie de Tunis, Faculté de médecine de Tunis, Université Tunis El Manar, Tunisie; 2 Service A, Centre de Maternité et de Néonatologie de Tunis, Faculté de Médecine de Tunis, Université Tunis El Manar, Tunisie; 3 Service de Réanimation, Centre de Maternité et de Néonatologie de Tunis, Faculté de Médecine de Tunis, Université Tunis El Manar, Tunisie

**Keywords:** Hémorragie du post-partum, ligature des artères hypogastriques, ligature vasculaire, capitonnage utérin, hystérectomie d´hémostase, Postpartum hemorrhage, Ligation of hypogastric arteries, Vascular ligation, Haemostatics brace suturing, Hemostasis hysterectomy

## Abstract

L'objectif était d'identifier les facteurs de succès de la ligature bilatérale des artères hypogastriques et évaluer sa place dans le traitement chirurgical des hémorragies du post-partum. Nous avons réalisé une étude rétrospective regroupant tous les cas d'hémorragie du post-partum ayant nécessité un traitement chirurgical entre Janvier 2008 et Décembre 2011. L'étude a concerné 88 patientes (0,47% du total des accouchements). L'atonie utérine était l'étiologie la plus fréquente (64,8 % des patientes). La ligature bilatérale des artères hypogastriques a été réalisée chez 81,8% des patientes. Quand elle était le premier geste chirurgical réalisé, son taux de succès était de 66%. Ce taux était variable en fonction de l'étiologie de l'hémorragie, de la présence ou non de troubles de l'hémostase et du temps écoulé entre le diagnostic et la réalisation du geste chirurgical. En cas atonie utérine, l'association d'une deuxième technique conservatrice lorsque la première était insuffisante, a permis d'arrêter le saignement dans 98% des cas. La ligature des artères hypogastriques est une technique chirurgicale efficace pour le traitement de l'hémorragie du post-partum. Son taux de succès est augmenté par sa réalisation précoce ainsi que son association à d'autres techniques conservatrices.

## Abstract (en)

The objective were to identify the success factors of bilateral hypogastric arteries ligation and to assess its role in surgical treatment of postpartum hemorrhages. We conducted a retrospective study of all the cases of postpartum hemorrhage requiring surgical treatment between January 2008 and December 2011. The study included 88 patients (0,47% of all births). Uterine atony was the most common etiology (64,8 % of patients). Bilateral ligation of the hypogastric arteries was performed in 81.8% of patients. When surgery was the first choice treatment, its success rate was 66%. This rate was variable depending on the etiology of hemorrhage, the presence or the absence of hemostasis disorders and the time between diagnosis and surgical treatment. In case of uterine atony, the association with a second conservative technique, when the first was inadequate, helped stop the bleeding in 98% of cases. Ligation of hypogastric arteries is an effective surgical technique for the treatment of postpartum hemorrhage. Its success rate has increased thanks to early implementation and to the association with other conservative techniques.

## Introduction

L'hémorragie du post-partum (HPP) est la première cause de mortalité maternelle en Tunisie et dans le reste du monde [1, 2]. Ses étiologies sont multiples notamment l'atonie utérine, les anomalies d'insertion placentaire, les ruptures utérines, les plaies cervico-vaginales et les coagulopathies acquises ou constitutionnelles [3]. La prise en charge chirurgicale de l'HPP prend une place prépondérante en cas d'échec du traitement médical et de la réanimation dans l'atonie utérine et les troubles de la coagulation. Elle est parfois nécessaire en complément des sutures dans d'autres étiologies tel que les plaies cervico-vaginales et les ruptures utérines. Différentes techniques chirurgicales conservatrices ont été décrites dans la prise en charge des HPP tel que la triple ligature de Tsirulnikov (TL) [4] , la ligature bilatérale des artères utérines [5], le capitonnage utérin [6, 7] et la ligature bilatérale des artères hypogastriques (LBAH) [8]. Cette dernière a été décrite pour la première fois par Sagarra dès 1960 [8]. Néanmoins, sa place exacte dans le diagramme de prise en charge des HPP a été peu étudiée, ainsi que l'utilité de lui associer d'autres techniques conservatrices. L'objectif de notre étude est d'identifier les facteurs de succès de cette technique, notamment le fait de lui associer d'autres techniques chirurgicales et d'évaluer la place de la LBAH dans la stratégie de prise en charge des HPP dans notre pratique quotidienne où l'accès aux techniques d'embolistation est limité.

## Méthodes

Il s'agit d'une étude rétrospective réalisée au Centre de Maternité et de Néonatologie de Tunis regroupant tous les cas d'hémorragie du post-partum ayant nécessité une prise en charge chirurgicale entre Janvier 2008 et Décembre 2011. Les cas d'HPP jugulés par un traitement médical exclusif ou survenant avant 28 semaines d'aménorrhée ont été exclus de l'étude. Dans notre centre, La prise en charge initiale en cas d'atonie utérine est standardisée. En cas d'échec d'un traitement médical initial par les ocytociques, un traitement médical de deuxième ligne par sulprostone (Nalador) est prescrit. Une prise en charge chirurgicale est indiquée en l'absence d'amélioration au bout de 20 minutes du début de la perfusion de sulprostone voir plus tôt en cas de saignement important. Dans les cas de rupture utérine ou de déchirures cervico-vaginales, on débute par une réparation chirurgicale des lésions. Concernant les anomalies d'insertion placentaire, la prise en charge est soit une hystérectomie d'hémostase (HH) d'emblée, soit une tentative de traitement conservateur selon la parité et l'état hémodynamique de la patiente. Dans les différents cas, les ligatures vasculaires et les plicatures utérines sont réalisées en complément si le saignement n'est toujours pas contrôlé.

Le traitement chirurgical est pratiqué par des chirurgiens gynécologues séniors. Les techniques pratiquées sont la TL, la LBAH, la plicature utérine selon B-Lynch modifié et l'HH. Le choix de la technique, l'ordre de réalisation et l'association des techniques chirurgicales varie selon l'étiologie de l'hémorragie, l'état hémodynamique de la patiente, sa parité et enfin les habitudes du chirurgien. Le « succès » est défini par l'arrêt du saignement avec survie de la patiente et l' « échec » par la persistance du saignement et la nécessité de recours à une autre technique ou le décès de la patiente. A noter que, nous ne disposons pas de sondes de Bakery dans notre centre et nous n'avons pas de possibilité d'embolisation radiologique pendant la garde et l'accès à cette technique est sinon difficile vu qu'elle n'est réalisée que par un seul radiologue dans la capitale. Les cas d'HPP ont été identifiés grâce à un système de codage et le recueil des données a été effectué à partir des dossiers d'hospitalisation et des comptes-rendus opératoires. Pour chaque patiente, nous avons recueilli les données anamnestiques, cliniques, para-cliniques et thérapeutiques ainsi que les complications secondaires. L'étude statistique a été faite avec le logiciel SPSS 20 (SPSS Inc., Chicago, IL). La comparaison entre les groupes a été faite par Test Khi 2 pour les variables qualitatives et le test de Student pour les variables quantitatives. Un p < 0,05 était considéré comme significatif.

## Résultats

**Figure 1:**
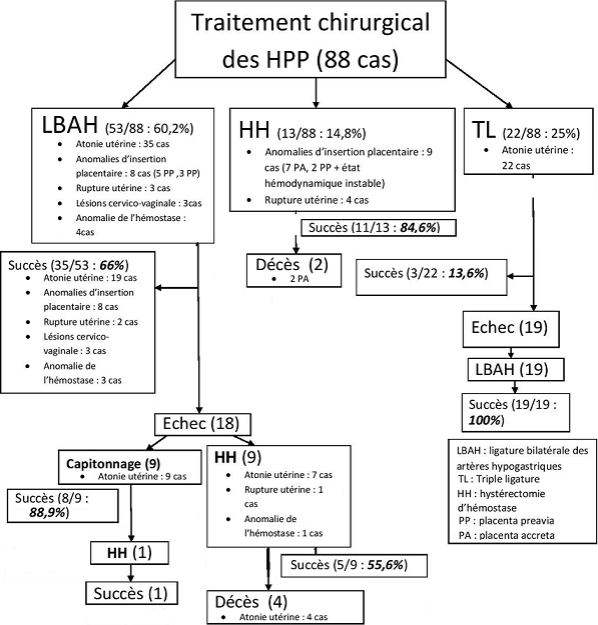
Séquence de réalisation des différentes techniques chirurgicales selon l’étiologie de l’HPP et leur taux de succès

Durant la période de l'étude 18 772 accouchements ont été recensés. Parmi ces accouchements, 450 se sont compliqués d'une hémorragie du post-partum (2,4% du total des accouchements) dont 88 ont nécessité le recours à des gestes chirurgicaux (0,47% du total des accouchements et 19,6% du total des hémorragies). Dans ce groupe de 88 patientes, la moyenne d'âge des patientes était de 31,5 ans (18-44). Parmi ces patientes, 82 % étaient âgées de moins de 35 ans et 35,2% étaient des primipares. L'accouchement s'est fait par césarienne dans 72,7% des cas dont 73,5% dans un contexte d'urgence. Outre l'accouchement par césarienne, les autres facteurs de risque d'HPP identifiés dans notre série étaient : l'anémie maternelle (37,5% des cas), l'utérus cicatriciel (27,3%), la prééclampsie (22,7%) et les anomalies d'insertion placentaire (19,3%). Les principaux facteurs de risque de l'HPP identifiés chez nos patientes sont résumés dans le Tableau 1. Concernant les étiologies de l'HPP, l'atonie utérine était l'étiologie la plus fréquente retrouvée chez 57 patientes (64,8 %), suivie par les anomalies d'insertion placentaire dans 17 cas (19,4%) dont 12 cas de placenta accréta et 5 cas de placenta prævia, la rupture utérine dans 7 cas (7,9% des cas), les lésions de la filière génitale dans 3 cas (3,4% des cas) et enfin les troubles de l'hémostase préexistant à l'accouchement dans 4 cas (4,5%). Les troubles de l'hémostase étaient secondaires à : un hématome rétroplacentaire (HRP) (2 cas), à une mort fœtale in utero (MFIU) non expliquée (1 cas) et à un traitement par antivitamine K (1 cas). Le temps moyen de diagnostic de l'HPP était de 25,6 minutes (10-210). Le diagnostic de l'HPP a été posé dans un délai inférieur à 30 minutes dans 73,9% des cas (65 patientes). Toutefois, au moment du diagnostic 7 patientes (6,8%) étaient en état de choc. La Figure 1 schématise la hiérarchie des différentes techniques chirurgicales utilisées selon l'étiologie de l'HPP ainsi que leur taux de succès. Au total, la LBAH a été réalisée chez 72 patientes (81,8% des cas). L'efficacité globale de la LBAH, seule ou associée à d'autres gestes conservateurs était de 86 ,1%.

**Tableau 1:** Facteurs de risque de l’hémorragie du postpartum retrouvés dans notre série

Facteur de risque	Nombre de cas (pourcentage)
**Anémie maternelle**	
**Sur distension utérine**	33 (37,5%)
Grossesse gémellaire	10 (11,4%)
Macrosomie	11 (12,5%)
Hydramnios	9 (10,2%)
**Utérus cicatriciel**	24 (27,3%)
**Préeclampsie**	20 (22,7%)
**Anomalies insertion placentaire**	
**Autres**	17 (19,3%)
ATCD d’hémorragie du post-partum	6 (6,8%)
Multiparité	6 (6,8%)
Travail rapide ou long	12 (8,1%)
Chorioamniotite	5 (5,7%)
Maturation cervicale	8 (9,1%)
Coagulopathie préexistante au travail	4 (4,5%)
Extraction instrumentale	5 (5,7%)

**Figure 2:**
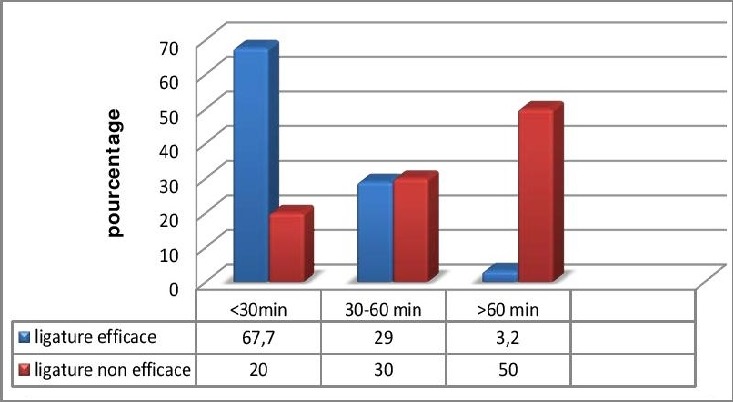
Taux de succès de la LBAH en fonction du temps écoulé entre le diagnostic de l’HPP et la réalisation du geste chirurgical

Le taux de succès de la LBAH d'emblée était de 66%. Ce taux était variable en fonction de l'étiologie de l'HPP : il était de 54,3% dans les atonies utérines, de 100% dans les anomalies d'insertion placentaire et les lésions cervico-vaginales, de 66,7% dans les ruptures utérines et de 75% dans les hémorragies secondaires à des troubles de l'hémostase. Cette différence entre ces différents taux de succès en fonction de l'étiologie était non significative (p=0,83). En étudiant individuellement le sous-groupe des patientes avec atonie utérine, on a noté que le taux de succès de LBAH seule était seulement de 54,3%. Dans ce même sous-groupe, en cas de persistance du saignement, une association d'un capitonnage utérin à une LBAH a permis d'atteindre un taux cumulé de succès de 96%. Une association d'une LBAH à une TL a permis d'atteindre un taux cumulé de succès de 100%. Au total, en cas d'atonie utérine, l'association d'une deuxième technique conservatrice lorsque la première s'est avérée insuffisante, a permis d'arrêter le saignement dans 98% des cas. Concernant la mortalité suite à une atonie utérine, celle ci était nulle si un capitonnage utérin a été réalisé après échec de la LBAH. Par ailleurs, toujours en cas d'atonie utérine, le taux de mortalité était de 57,1% lorsque une HH a été réalisée d'emblée après échec de la LBAH. Le taux de succès de la LBAH était aussi variable en fonction du temps écoulé entre le diagnostic de l'HPP et la réalisation du geste chirurgical. Ainsi, ce taux était de 67 ,7% lorsque la ligature était réalisée dans les 30 minutes et passait à seulement 3,2% lorsque celle-ci était réalisée après 60 minutes (Figure 2). Cette différence était significative (p<0,0001). Ce taux était de 57,1% (4/7 cas) en cas de troubles de l'hémostase contre 89% (58/65cas) en dehors de ces troubles (p=0,02). Nous avons noté 15 complications maternelles dans notre série : deux plaies de la veine iliaque interne au cours des LBAH, deux plaies vésicales dans un contexte de rupture utérine complexe, 2 cas thrombophlébites du membre inférieur, 2 cas d'embolies pulmonaires, 4 cas d'œdème aigue du poumon et 3 cas d'infection de la paroi abdominale. L'évolution était favorable dans tous ces cas, notamment les deux cas de plaies de la veine iliaque interne qui ont été réparées par des sutures simples. Le taux de mortalité maternelle par HPP était de 0,3‰ (6 patientes). En cas d'hémorragie déclarée, le risque de décès était de 1,3%. Ce risque passait à 6,8% en cas d'HPP nécessitant un traitement chirurgical. Les caractéristiques des six patientes décédées sont résumées dans le Tableau 2. En post opératoire immédiat, 55 patientes (62,5% des cas) ont été transférées au service de réanimation. La durée moyenne de séjour en réanimation était de 32,5 heures (12-192).

**Tableau 2:** Les caractéristiques des cas de décès maternel dans notre série

	Age	Parité	Mode d’accouchement	Etiologie de l’HPP	Délai de diagnostic de l’HPP	Délai de réalisation LBAH	Heure de décès par rapport au diagnostic
1	30	3	Césarienne en urgence (utérus bicicatriciel en travail)	Atonie utérine	15 mn	20 mn	H 6
2	40	4	Césarienne en urgence (utérus tricicatriciel + chorioamniotite)	Placenta accreta	10 mn	-	H 6
3	39	4	Césarienne en urgence (placenta preavia hémorragique)	Placenta accreta	10 mn	-	H 8
4	44	3	Accouchement instrumental par voie basse	Atonie utérine	40 mn	45 mn	H 4
5	35	2	Césarienne en urgence pour patiente cardiaque	Atonie utérine	10 mn	20 mn	H 4
6	38	3	Césarienne en urgence pour grossesse gemellaire sur utérus cicatriciel	Atonie utérine	10 mn	15 mn	H 8

## Discussion

Notre étude a permis de mettre en évidence que, même dans les centres à fort débit obstétrical, le traitement chirurgical des HPP n'est pas standardisé. Ce volet chirurgical de la prise en charge et la stratégie optimale à adopter prend encore plus de l'importance dans les pays en voie de développement où l'accès à l'embolisation radiologique est difficile et où les Sondes de Bakery sont le plus souvent non disponibles. Le choix de la technique initiale et de l'association secondaire à d'autres procédures dépend alors, essentiellement de l'étiologie, de l'état clinique de la patiente, de sa parité et enfin de l'expérience et des habitudes du chirurgien. Dans notre série, la LBAH a été réalisée de première intention dans 60,2% des cas et elle a été réalisée au total chez 81,8 % de l'ensemble des patientes. Ces taux élevés peuvent être expliqués par le fait que les gynécologues de notre équipe sont rodés à cette technique et qu'ils préfèrent la réaliser tôt pour augmenter ses chances de succès. L'efficacité de la LBAH de première intention varie dans les études de 42 à 100% [9–20] (Tableau 3). Ce taux était de 66,03% dans notre série. En fait, le taux de succès varie selon l'étiologie de l'HPP. Ainsi, en cas d'atonie utérine, le taux de succès était de 40% dans la série de Clark et al [9] et de 89% dans l'étude de Ledee et al [10]. Ce taux de succès varie entre 25% et 86% dans les cas d'anomalies d'insertion placentaire et entre 0% et 100% dans les cas de ruptures utérines (Tableau 3). Le nombre limité des cas inclus dans ces différentes études et leurs caractères hétérogènes est probablement à l'origine de ces grandes discordances. La plus large série de LBAH a été rapportée par Koné et al (134 cas) [11]. Le taux de succès de la LBAH était de 87%. Ce taux en cas d'atonie utérine, d'anomalies d'insertion placentaire et de rupture utérine était respectivement de 79%, 82% et 87%. Dans notre étude, ces taux étaient de 54,3%, 100% et 66,7%. En cas de coagulopathie préexistante à l'accouchement, le taux de succès de la LBAH d'emblée dans notre série était de 75%. Joshi et al. ont rapporté un taux de succès dans cette situation de 90,9% [12].

**Tableau 3:** Éfficacité de la ligature première des artères hypogastriques dans les hémorragies du post-partum dans différentes séries publiées

Auteur, année	Nombre de patientes	Arrêt du saignement n(%)	Taux de succès par pathologie (%)		
			AT	AIP	RU
Clark et al, 1985 [9]	19	8 (42)	40		
Evans et al, 1985 [11]	14	6(42)		50	
Thavarasah et al, 1989 [12]	14	10 (72		25	0
Chattopadhyay et al, 1990 [13]	29	19 (65)	50	75	
Likeman et al, 1992 [14]	15	15 (100)			
Das et al, 1998 [15]	15	14 (75)	75		
Ledee et al, 2001 [10]	48	43 (89)	89	83	
Joshi et al, 2006 [16]	88	51 (61)	64	86	21
Koné et al, 2009 [17]	159	134 (87)	79	82	87
Camuzcuoglu et al, 2010 [18]	24	18 (75)			100
Unal et al, 2011 [19]	58	51 (87,9)	55	100	67
Notre série	53	35 (66)			

AT : atonie utérine ; AIP : anomalie d’insertion placentaire ; RU : rupture utérine

Source : Haumonté et al [20]

Par ailleurs, l'efficacité varie aussi en fonction du délai de réalisation du geste. Un délai court entre le diagnostic de l'hémorragie et la LBHA serait un facteur de succès de la technique [13]. Dans la série de Chattopadhyay, le temps moyen entre le diagnostic de l'HPP et la réalisation de la LBAH était de 20 minutes en cas de succès et de 55 minutes en cas d'échec [14]. Dans celle de Koné, le délai moyen dans les groupes succès et échec, était respectivement, de 50 et 96 minutes (p<0,0001) [11]. Ce facteur a été aussi trouvé dans notre étude puisque le taux de succès des LBAH était 67 ,7% si celle ci était réalisée dans les 30 minutes et seulement de 3,2% après 60 minutes (P<0,0001). Le taux de succès était aussi significativement diminué dans notre série en cas de présence d'un état de choc ou un trouble de l'hémostase. Koné a lui aussi identifié dans sa série ces deux facteurs comme étant des facteurs significatifs indépendants d'échec [11]. En effet, en plus de l'aggravation du saignement présent, ces conditions sont souvent responsables de difficultés techniques lors de la réalisation de la LBAH tel qu'un saignement inhabituel lors de la dissection du rétropéritoine ou des difficultés à identifier l'artère hypogastrique en cas de collapsus. En cas d'atonie utérine, nous avons noté une amélioration des taux de succès si la LBAH était associée à d'autres techniques chirurgicales conservatrices. Ainsi, le taux de succès en cas de LBAH seule était de 54,28%. En cas de persistance du saignement, une association à un capitonnage utérin a permis d'atteindre un taux de succès de 96%. Une association d'une TL à une LBAH a permis d'atteindre un taux cumulé de succès de 100%. Au total, en cas d'atonie utérine, l'association d'une deuxième technique conservatrice en cas d'échec de la première (notamment une LBAH suivie d'un capitonnage ou une TL suivie d'une LBAH) a permis d'atteindre un taux de succès global de 98% . L'efficacité de l'association de la LBAH à la plicature utérine selon B-Lynch dans l'atonie utérine n'a été rapportée que sous forme de cas cliniques [15–17]. Dans notre série, on note que 4 cas de décès secondaires à une atonie utérine faisaient suite à une HH qui a suivie directement la LBAH. Ceci ne pourrait pas être expliqué par le fait qu'il s'agissait de cas d'atonies utérines diagnostiquées tardivement puisque le délai moyen de diagnostic était de 18,7 minutes (10-40). La décision de procéder à une HH a été plutôt motivée par la multiparité des patientes concernées. Le taux de mortalité élevé devant une telle conduite incite à repenser le recours à l'HH au profit de l'association de techniques conservatrices si possible. Ces associations étant plus rapides à réaliser et entrainent moins de pertes sanguines et moins de décollements pendant leur réalisation. Les complications liées directement à la LBAH peuvent être à type de plaie de la veine iliaque ou de l'artère iliaque externe, de lésion urétérale ou de lésion nerveuse périphérique [13]. Le taux de complication varie dans la littérature de 0 à 13% en varie en fonction de l'expérience des l'opérateurs [13]. Seuls deux cas de plaie de la veine iliaque ont été rapportés dans notre série avec une évolution favorable après sutures. Le retentissement de ces différentes techniques chirurgicales sur la fertilité ultérieure a été étudié par Doumouchtsis et al dans une revue de la littérature publiée en 2014. Ainsi, après un traitement chirurgical conservateur d'une HPP, 77,9% des patientes désireuses de grossesse ont réussi à concevoir [18].

## Conclusion

La maitrise des différentes techniques chirurgicales conservatrices en cas d'HPP est indispensable. Le choix de l'ordre de réalisation et des éventuelles associations doit prendre en considération plusieurs facteurs. Ainsi, le résultat d'une LBAH peut être optimisé par le choix de l'indication et l'association à d'autres techniques chirurgicales conservatrices comme il peut être limité par un retard de réalisation. Afin d'optimiser les résultats de la LBAH dans la prise en charge chirurgicales des HPP on peut appuyer certaines conduites : en Réalisant celle-ci le plus tôt possible, encore plus avant l'installation d'un état de choc ou de troubles de l'hémostase. Devant une atonie utérine, et en cas d'échec d'une première technique conservatrice, il faut penser à associer, si l'état clinique de la patiente le permet, une autre technique conservatrice (TL, plicature utérine) plutôt que de procéder à une HH. Cette stratégie permet de juguler l'hémorragie dans 98% des cas.

### Etat des connaissances actuelles sur le sujet

- Intérêt de la ligature des artères hypogastriques dans la prise en charge chirurgicale de l'hémorragie du post-partum et notamment dans l'inertie utérine.
- Intérêt des différentes techniques chirurgicales conservatrices dans la prise en charge chirurgicale de l'hémorragie du post-partum (triple ligature de Tsirulnikov, ligature des artères utérines, capitonnage utérin et ligature des artères hypogastriques).

### Contribution de notre étude à la connaissance

- Intérêt de l'association de 2 techniques chirurgicales conservatrices dans le traitement chirurgical de l'inertie utérine (notamment ligature des artères hypogastriques + plicature utérine ou triple ligature de Tsirulnikov + ligature des artères hypogastriques).
